# Intersectionality in nursing research: A systematic review

**DOI:** 10.1002/nop2.2021

**Published:** 2023-10-05

**Authors:** Elin Siira, Karolina Linden, Sara Wallström, Ida Björkman

**Affiliations:** ^1^ School of Health and Welfare Halmstad University Göteborg Sweden; ^2^ Institute of Health and Care Sciences Gothenburg University Göteborg Sweden; ^3^ Centre for Person‐Centred Care GPCC Gothenburg University Göteborg Sweden

**Keywords:** intersectionality, nursing research, systematic review

## Abstract

**Aim:**

This systematic literature review aimed to identify, appraise and synthesize available research studies that apply intersectionality in nursing research.

**Design:**

Systematic review.

**Data Sources:**

Empirical and theoretical nursing studies published before February 2022 were identified from the PubMed and CINAHL databases. Studies were eligible for inclusion if they substantially covered the topics of intersectionality and nursing, had undergone peer‐review, and were written in English.

**Review Methods:**

The PRISMA 2020 statement for reporting systematic reviews was used to report findings. The Joanna Briggs Institute Critical Appraisal tools were used to assess the quality of the included research studies.

**Results:**

Out of 331 identified studies, 60 studies were substantially about nursing and intersectionality, and were included in the review. There are a myriad of ways that the concept of intersectionality has been adopted in nursing research. Furthermore, there was great heterogeneity in the definition and application of the concept of intersectionality, and only a few studies were empirical.

**Conclusion:**

There is a need for robust and clear framing of how the concept of intersectionality is defined and understood in nursing research. There is also a need for more empirical research effectively adopting the concept of intersectionality to enhance our understanding of how health inequities operate within the field of nursing.

**No Patient or Public Contribution:**

No patients, service users, caregivers or members of the public were involved in this work.

## 1 INTRODUCTION

There have been calls to move beyond a patient‐dyad perspective and individual‐focused frameworks in nursing to address peoples' social context, including structural inequalities that contribute to ill health (Tengelin et al., 2019; Thurman & Pfitzinger‐Lippe, 2017). Pervasive health inequities exist within and between countries and can be traced to an unequal distribution of power, resources and access to services that is sometimes referred to as social determinants of health (Marmot et al., 2008). Health outcomes at the individual level are thus linked to unjust social policies, economic planning and politics, and the World Health Organization (WHO) has identified, for example, racism as one social determinant of health inequities (Marmot et al., 2008; World Health, 2010). There are multiple examples of groups who are disadvantaged because of their race, gender, age, disability or socioeconomic status (Hosseinpoor et al., 2015). These social categories also affect access to and experiences of healthcare services (Marmot et al., 2008). The literature on social determinants of health has faced criticism for concentrating on a singular aspect of health inequality, for example, gender or class, while disregarding others. Thereby limiting the understanding of how these factors interact. Introducing intersectionality as a framework for analysing health inequalities is argued to provide a much more nuanced and precise understanding of their mechanisms (Sen et al., 2009). Intersectionality and its focus on several social categorizations has become an indispensable and extensively used framework for public health research (Bauer, 2014; Bowleg, 2021; Hankivsky, 2012; Viruell‐Fuentes et al., 2012) Over the last decade, nursing research has increasingly applied intersectionality theory to analyse and understand health inequalities (De Sousa & Varcoe, 2022). Although intersectionality is widely regarded as a useful concept, with nearly universal applicability, which in part explains its widespread success (Davis, 2008), its value in nursing research largely depends on how ideas of intersectionality are employed and defined.

## 2 BACKGROUND

### 2.1 Intersectionality and nursing

According to the Oxford English Dictionary, an ‘intersection’ is where two things cross or intersect (OED Online, ‘*intersection, n*.’, 2022a), while ‘intersectionality’ refers to a theoretical approach from sociology based on the interconnected nature of social categorizations such as race, class and gender, which create overlapping and interdependent systems of disadvantage or discrimination (OED Online, ‘intersectionality, n.’, 2022b). The concept of intersectionality emerged in academic discourse during the late 1980s through the individual works of legal scholar Kimberlé Crenshaw (1989, 1991) and sociologist Patricia Hill Collins (1986). However, its foundational ideas originated in the 1960s and 1970s in the United States and were influenced by the Civil rights, women of color, Black power, Chicano liberation, Red power and Asian‐American movements, as well as feminist lesbian organizations such as the Combahee River Collective (Bilge, 2013; Collins & Bilge, 2016; Dhamoon, 2011; Hancock, 2016). Apart from its theoretical and scholarly contributions, the concept has been closely linked to activism and the empowerment of black women. For example, combating violence against women of color constitutes central focus of intersectionality activism. Therefore, the concept of intersectionality serves as a means to both comprehend and create strategies to combat inequalities (Collins & Bilge, 2016). Despite its strong connections to feminism and gender studies, intersectionality does not have to focus solely on gender (Collins & Bilge, 2016). The intersection that is most frequently analysed is the one that involves race, class and gender (Dhamoon, 2011).

In academic writing, intersectionality is often presented as a constantly evolving research paradigm or a framework (Dhamoon, 2011). What actually constitutes an intersectional analysis remains a topic of debate, but it can be broken down into six fundamental ideas (Collins & Bilge, 2016):

- Complexity
- Power
- Inequality
- Relationality
- Social context
- Social justice 

Regarding *complexity*, intersectionality prompts a view that gender‐related inequality and race‐related inequality are not separate issues. This view involves attending to and making visible the diversity within groups such as diversity within the group of women regarding social class, race or sexual orientation (Collins & Bilge, 2016). Marginalized groups often face skewed representations in societal discourse, and it is crucial to acknowledge and incorporate other perspectives on what it entails to be a black woman, for instance (Hancock, 2016). When it comes to issues of *power*, a postmodern, ‘Foucauldian’, understanding is prevalent within intersectionality. This suggest that power is a constantly shifting and relational concept rather than being attributed solely to individuals or groups (Hancock, 2016). Individuals can simultaneously occupy the roles of oppressor and oppressed depending on the circumstances and therefore, there is no concept of ‘true victims’ (Collins, 2000). As an example, the nurse may hold power in their interactions with a patient, yet not in the professional hierarchy of the healthcare institution. These dimensions of power are interrelated but separate and relate to interpersonal, disciplinary, hegemonic and structural domains (Collins, 2000). The first domain addresses how people are positioned within social interactions, such as nurses' interactions with patients. The interpersonal dimension of power is central to nursing and it is often stated that the relationship between nurse and patient is asymmetrical (Kristoffersen & Friberg, 2017). The disciplinary and structural domains can also relate to nursing through the power of healthcare institutions in deciding what is ‘normal’ versus ‘deviant’ as well as being part of the wider web of the large‐scale institutions that reproduce subordination (Collins, 2000). The dynamics of power in all these domains are relevant to health (Collins, 2022). *Inequality* refers to individuals having different positions in the social hierarchy and therefore unequal access to social and economic resources and opportunities. This is a result of unjust social processes and systems (Collins & Bilge, 2016). *Relationality* refers to the interplay between various sorts of social inequalities and systems of power and accounts for multiple identities within the interpersonal domain. Furthermore, it rejects an either/or thinking in favour of relatedness between social positionings (Collins & Bilge, 2016). For example, a nurse can also be a woman, an immigrant and a mother. *Social context* plays an important role in shaping people's experiences, influenced by the time and place in which they live. *Social justice* refers to promoting fairness in the allocation and availability of social and economic resources and opportunities in society occur (Collins & Bilge, 2016). Combating inequality in care, the social justice mandate and a focus on peoples' social context have been suggested as fields where nursing can make advances (Tengelin et al., 2019).

Finally, there are different types of analytical foci within intersectional research such as identities, categories of difference, processes of differentiation and systems of domination. For example, black is an identity belonging to the category race that arises through racialization, with the racism as the linked system of domination. Other systems of domination are sexism, ableism, classism, heterosexism, etc. (Dhamoon, 2011).

There is extensive literature from different disciplines on the potential and pitfalls of applying the ideas of intersectionality in research (Anthias, 2013; Bilge, 2013; Collins, 2015; Davis, 2008; Knapp, 2005). Apart from scholarship, intersectionality is also prevalent within popular culture as a ‘buzz word’ or meme (Hancock, 2016). The literature contends that the popularity of intersectionality also creates issues, including misinterpretation, displacement and disarticulation or presenting it as a formula without regard for the concept's origins (Bilge, 2013; Knapp, 2005; Moradi et al., 2020). There is also some ambivalence as to how intersectionality, as a theoretical concept, can be incorporated into empirical research (Hancock, 2016).

### 2.2 Borrowed theory in nursing

Drawing on feminist standpoint theory, Risjord (2010) contends that nursing research occupies a distinct position as an ‘oppressed role’ that enables access to knowledge that is not readily available from other perspectives. Since intersectionality theory was not formulated within the nursing discipline it may be regarded as a ‘borrowed theory’. A borrowed theory is a theory, which is derived from disciplines outside of nursing (such as sociology, psychology or physiology), and adopted into the nursing discipline (Risjord, 2010). Despite some nursing theorists' concerns about the potential negative impact of borrowed theories on nursing research, they have nonetheless become an integral aspect of nursing research (Risjord, 2010). Borrowed theories, including intersectionality theory, require adaption to fit the nursing context and the defining characteristics of nursing research. Nursing research aims to address questions that are relevant to nursing practice in a direct or indirect manner (Forss et al., 2013). The idea of borrowed theory (Risjord, 2010) also implies that the adoption of intersectionality theory within nursing will have a substantial influence on both nursing research and the conceptualization of intersectionality. Therefore, the notion of borrowed theory in nursing emphasizes the interaction between disciplines and concepts that transpires when disciplinary boundaries are crossed, and concepts or theories are employed in novel knowledge contexts. This process of adoption will have an impact on the potential and possibilities of intersectionality in nursing. Thus, a critical examination of the practical application of intersectionality and its outcomes, rather than only its potential benefits, is necessary.

This systematic literature review aims to identify, appraise and synthesize available research studies that apply intersectionality in nursing. We examine the characteristics of nursing research that apply intersectionality, definition of intersectionality in this research and the rationale for its application. Additionally, we consider the implications of applying intersectionality for nursing education, scholarship and practice as identified by the authors. It has been argued that incorporating intersectionality into research can enhance theoretical and methodological work rather than practice (Moradi et al., 2020). Nursing is a practice‐based discipline, and thus, acknowledging practice is critical to it. Our overview will give the reader a sense of the many ways to conduct research using intersectionality theory in nursing. Thereby, this paper also provides a toolbox for nursing scholars aiming to adopt the concept of intersectionality in their research.

## 3 THE REVIEW

### 3.1 Aim

This systematic literature review aims to identify, appraise and synthesize available research studies that apply intersectionality to nursing. For this purpose, we aim to answer the following research questions:

- What are the characteristics of nursing research applying intersectionality?
- How is intersectionality defined in nursing research?
- What are the arguments for applying intersectionality in the context of nursing research?
- What are the author‐identified implications of applying intersectionality in nursing research?

### 3.2 Design

A systematic review (Grant & Booth, 2009) was undertaken to identify, appraise and synthesize available research studies that apply intersectionality to nursing. Guidelines on the general steps required for a systematic review by the Joanna Briggs Institute (JBI) guided the design of the review. These steps were: (1) formulating a review question, (2) defining inclusion/exclusion criteria, (3) identifying studies through searching, (4) selecting studies for inclusion, (5) assessing the quality of included studies, (6) extracting data, (7) analysing included studies and (8) interpreting and reporting results (see Aromataris & Munn, 2020). The review followed the Preferred Reporting Items for Systematic Reviews and Meta‐Analyses (PRISMA 2020) guidelines (Page et al., 2021) (see Table S1).

### 3.3 Search methods

Research published before February 2022 was identified within the PubMed and The Cumulative Index to Nursing and Allied Health Literature (CINAHL) databases. The two databases PubMed (which includes citations for literature from MEDLINE) and CINAHL were selected as they encompass studies specifically related to nursing. No publication date limitations were considered as we wished to identify any available research articles that applied intersectionality in nursing.

Given the breadth of nursing research, we anticipated that studies would be heterogeneous in terms of focus, research purposes, methods, participants and context. Furthermore, we anticipated that appropriate studies would be scarce. To account for broad indexing for intersectionality and nursing, we used intersectional* and nurs* as search terms. For details, see Table S2. Search strategy and database selection were determined in consultation with a medical librarian who specialized in information science at the Biomedical Library at Gothenburg University in Sweden.

### 3.4 Study selection process

A study was eligible for inclusion if it was (a) substantially about intersectionality and nursing, (b) peer‐reviewed and (c) written in English. Studies were not excluded based on study design, for example, both empirical and theoretical studies were included, but editorials and commentaries were deemed ineligible because they are not peer‐reviewed. As we aimed to identify available research articles that apply intersectionality in nursing, theses, book chapters and grey literature were excluded. First, at least two of the authors independently screened titles and abstracts of the retrieved sources. Studies were later screened in full text if they (a) matched the inclusion criteria or if (b) the relevance of a study was unclear when screening its abstract/title. We also (c) screened all studies that were substantially about intersectionality and published in a nursing journal in full text, even if the abstract was not substantially about nursing. The latter was done to assure all articles with potential relevance for nursing were identified. Studies that met the inclusion criteria when screened in full text were included in the review. To screen for additional studies, we manually searched the reference list of the included studies. No additional studies were identified in this way. Disagreements concerning the screening process were discussed among the authors until a consensus was reached.

### 3.5 Quality appraisal

The quality appraisal was conducted to assess the overall methodological quality of the included studies, not as an inclusion/exclusion criterion. At least two authors independently assessed the studies' quality using the critical appraisal tools from the JBI (2022). According to the article's design, the appropriate checklist was used: qualitative research (Lockwood et al., 2015), text and opinion papers (McArthur et al., 2015), systematic reviews, meta‐synthesis (Aromataris et al., 2015), cross‐sectional studies (Moola et al., 2020), case reports or quasi‐experimental studies (Tufanaru et al., 2020). Examples of questions included in the critical appraisal tools are: (1) Were the criteria for inclusion in the sample clearly defined? (2) Is there congruity between the research methodology and the research question or objectives? and (3) Is the review question clearly and explicitly stated? Disagreements were discussed until a consensus was reached. An overview of which tool was used for each article and their scores can be found in the appendix for Table S3–S8.

### 3.6 Data extraction

Data relevant to answering the research questions were extracted independently by at least two of the authors using a data‐extraction sheet. The data‐extraction sheet was first piloted with five studies to ensure the usefulness of the groupings for answering the research questions. The sheet included: (1) study characteristics (i.e. publication year, country, author(s) and title), (2) methods and focus of the studies (i.e. aim, patient group/group/phenomenon of interest, setting, design/method), (3) features of the studies concerning applying intersectionality (i.e. definition of intersectionality, arguments for applying intersectionality) and (4) author‐identified implications for nursing education, scholarship and practice. All extracted data were entered into Microsoft Excel according to these specific areas in order to construct a table for analysis.

### 3.7 Data synthesis

The included studies comprised a diversity of study designs. Thus, it was not possible to conduct a meta‐analysis of the included studies. We, therefore, conducted a narrative synthesis. The extracted data on study characteristics and methods and focus were deductively grouped and clustered to answer research question 1. To answer research questions 2–4, we first grouped and clustered the extracted data on features of the studies concerning the concept of intersectionality. Second, we deductively established the main themes related to the research questions from each of the studies and juxtaposed it to identify patterns in the extracted material. At least two of the authors were involved in the data synthesis and disagreements were discussed until a consensus was reached.

## 4 RESULTS

**FIGURE 1:**
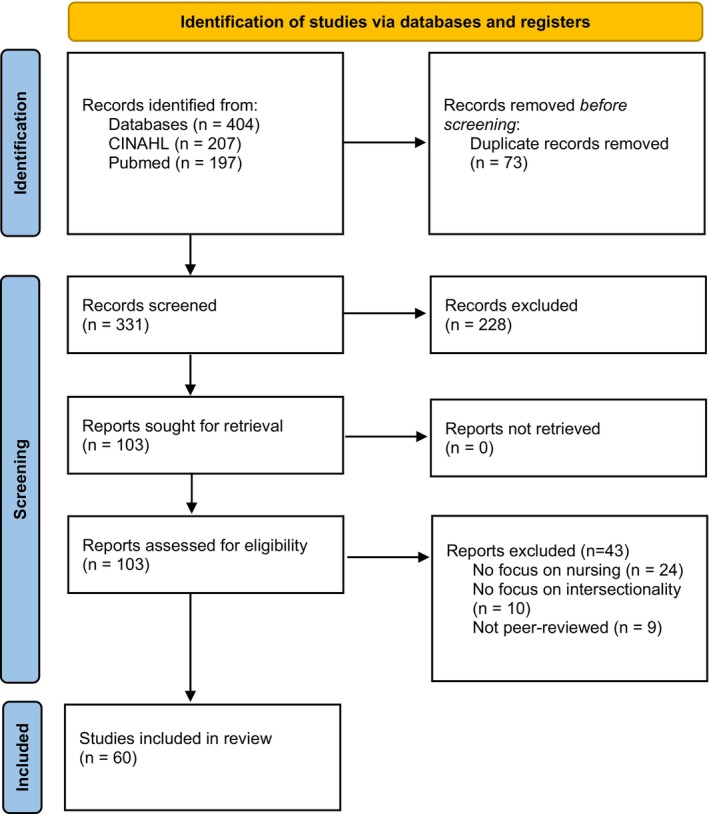
Flow chart. Search and retrieval results. *From*: Page MJ, McKenzie JE, Bossuyt PM, Boutron I, Hoffmann TC, Mulrow CD, et al. The PRISMA 2020 statement: an updated guideline for reporting systematic reviews. *BMJ* 2021;372:n71. doi: 10.1136/bmj.n71.

In total, 404 citations published between September 1997 and February 2022 were identified. After removing any duplicates (*n* = 73), 331 records remained. 228 records were excluded with reason by screening titles and abstracts. Thereafter, the full text of 103 records were screened and assessed for eligibility. An additional 43 articles were excluded with reasons after a full‐text review. In total, 60 articles were included in the review (Figure 1).

### 4.1 Characteristics of studies

The general characteristics (i.e. publication year, country, aim, patient group/group/phenomenon, setting, design/method) of the studies are outlined in Table 1. Most studies were published after 2010 (*n* = 55). A few were published between 2000 and 2009 (*n* = 4) and one was published before 2000. The most common country of origin was the United States (*n* = 26), followed by Canada (*n* = 22). Other countries of origin were Sweden (*n* = 5), the United Kingdom (*n* = 3), New Zealand (*n* = 2), Australia (*n* = 1) and Brazil (*n* = 1). The most common design was theoretical (*n* = 27), followed by empirical with a qualitative approach (*n* = 19), empirical with a quantitative approach (*n* = 3) and different types of reviews (*n* = 8). Two studies were quasi‐experimental (Henriquez et al., 2019; Höglund et al., 2016) and one study was a case report (McCall & Lauridsen‐Hoegh, 2014).

**TABLE 1:** Characteristics of included studies sorted per year of publication from oldest to most recent.

Year	Country	Author & title	Aim	Patient group/group/phenomenon of interest	Setting	Design
1997	US	Henderson *Intersecting Race and Gender in Feminist Theories of Women's Psychological Development*	Critique and develop psychoanalytic theory on women's psychological development and discuss implications for mental health nursing.	Women and race	Mental health nursing	Theoretical
2004	Canada	Guruge & Khanlou *Intersectionalities of influence*: r*esearching the health of immigrant and refugee women*	Theorize and operationalize the concept [intersectionalities of influence] in mental health promotion research with immigrant and refugee women	Women with immigrant/refugee status	Mental health nursing	Theoretical
2009	UK	Jones et al. *Caribbean nurses migrating to the UK*: *a gender‐focused literature review*	Explore the significance of gender in the context of the migration of nurses from the Caribbean region to the United Kingdom.	Caribbean Nurses migrating to the United Kingdom	Work environment of nurses	Review
2009	US	Kelly et al. *Integrating Intersectionality and Biomedicine in Health Disparities Research*	Discuss feminist intersectionality and biomedical approaches, their contributions to research, and integration in the research process	Latino women who experience intimate partner violence	All nursing	Theoretical
2010	Canada	Caxaj & Berman *Belonging Among Newcomer Youths Intersecting Experiences of Inclusion and Exclusion*	Explore newcomer youths' gendered, racialized, and class experiences of inclusion and sense of belonging in their country of resettlement	Newcomer youths	All nursing	Empirical – qualitative
2010	Canada	Van Herk et al. *Identity matters*: *Aboriginal mothers' experiences of accessing health care*	Explore the implications of how service providers' perceptions of Aboriginal women's identities influence their experiences of care access	Aboriginal mothers	Community nursing	Empirical – qualitative Secondary analysis
2011	Canada	Benbow et al. *Mothers with mental illness experiencing homelessness*: *a critical analysis*	To examine existing oppression and ongoing resistance in the lives of homeless mothers with mental illness and to learn from these women what is conducive to their health.	Mothers with mental illness experiencing homelessness	Mental health nursing	Empirical‐qualitative
2010	Canada	Guruge et al. *Intimate male partner violence in the migration process*: *intersections of gender, race and class*	Report Sri Lankan Tamil Canadian immigrants' perspectives on factors that contribute to IMPV in the postmigration context.	Intimate male partner violence among immigrants	All nursing	Empirical – qualitative
2011	US	Rogers & Kelly *Feminist intersectionality*: *Bringing social justice to health disparities research*	Provide an ethical approach to health disparities research that simultaneously describes and seeks to eliminate health disparities.	Health disparities research	All nursing	Theoretical
2011	US	Kelly *Theories of Intimate Partner Violence*: *From Blaming the Victim to Acting Against Injustice Intersectionality as an Analytic Framework*	Describe and expose the complexity of life experiences within intersecting oppressions in the context of intimate partner violence	Women who experience intimate partner violence	All nursing	Theoretical
2011	US	Shade et al. *A Conceptual Model of Incarcerated Adolescent Fatherhood*: *Adolescent Identity Development and the Concept of Intersectionality*	Present a model to guide research on adolescent fatherhood, in the context of incarceration, and suggest clinical intervention to improve outcomes for the young father and his child	Adolescent, incarcerated fathers and their children	Mental health nursing	Theoretical
2011	Canada	Van Herk et al. *Examining our privileges and oppressions*: *incorporating an intersectionality paradigm into nursing*	Discuss the hegemony of the white, middle‐class perspective in nursing	Oppression and privilege within the nursing profession and practice	All nursing	Empirical ‐– qualitative
2012	Canada	Guruge *Intimate Partner Violence*: *A Global Health Perspective*	Summarize literature on health consequences, costs, prevalence, risk factors, perceptions, and manifestations of intimate partner violence, and women's responses to it	Women who experience intimate partner violence	All nursing	Review
2012	Sweden	Saarnio et al. *Power relations in patients experiences of suffering during treatment for cancer*	Examine how patients who have cancer experience suffering in the context of power relations	Patients undergoing cancer treatment	Oncology nursing	Empirical – qualitative
2013	Canada	Chulach & Gagnon *Rethinking the experience of HIV‐positive refugee women in the context of pregnancy*: *using an intersectional approach in nursing*	Briefly overview the origins and evolution of intersectionality, describe levels of analysis and usefulness for nursing	Pregnant refugee women living with HIV	All nursing	Theoretical
2013	Canada	McCall & Lauridsen‐Hoegh *Trauma and cultural safety*: *providing quality care to HIV‐infected women of aboriginal descent*	Describe the case of a 52‐year‐old, HIV‐infected Aboriginal woman in relation to colonization, intersectionality, post‐traumatic stress disorder, depression, revictimization, and substance use	Women of aboriginal descent living with HIV	All nursing	Case report
2013	Canada	Giesbrecht et al. *Identifying socio‐environmental factors that facilitate resilience among Canadian palliative family caregivers*: *A qualitative case study*	Identify socio‐environmental factors that contribute to palliative family caregiver resilience in the Canadian homecare context	Palliative family caregivers	Home care nursing	Empirical – qualitative Secondary analysis
2013	US	Green *Application of the Self Care Deficit Nursing Theory*: *The Community Context*	Establish the usefulness of Orem's Self‐Care Deficit Nursing Theory in application to disabled children in the school setting and link it to social determinants of health and intersectionality	Schoolchildren with disabilities	School nursing	Theoretical
2014	Canada	Choby & Clark *Improving health*: *structure and agency in health interventions*	Critique liberal individualist assumptions of health and propose steps for critical realist intersectional interventions research	Structure and agency in health interventions	All nursing	Theoretical
2014	US	Caiola et al *Using an Intersectional Approach To Study the Impact of Social Determinants of Health for African American Mothers Living with HIV*	Broaden the discussion about conceptual approaches which can be used to address health and health inequities in nursing	African American mothers living with HIV	All nursing	Theoretical
2014	Sweden	Holmgren et al. *Intersectional perspectives on family involvement in nursing home care*: *rethinking relatives' position as a betweenship*	To understand, in the context of intersectional theory, the roles of family members in nursing home care	Relatives' involvement in nursing homes	Nursing homes	Empirical – qualitative
2014	Canada	Reimer‐Kirkham *Nursing Research on Religion and Spirituality Through a Social Justice Lens*	Critically analyse nursing discourses on religion and spirituality	Religion and spirituality in nursing	All nursing	Theoretical
2016	Sweden	Cuesta & Rämgård *Intersectional perspective in elderly care*	Contribute to staff well‐being in elderly care by asking in what way an intersectional perspective can contribute to increased knowledge of power structures	Employees at a nursing home with immigrant status	Work environment of nurses	Empirical – qualitative
2016	US	Hall & Carlson *Marginalization A Revisitation With Integration of Scholarship on Globalization, Intersectionality, Privilege, Microaggressions, and Implicit Biases*	Examine and discuss recent scholarship on marginalization and building the knowledge base in nursing while respecting diversity.	All marginalized groups/marginalization	All nursing	Theoretical
2016	Sweden	Höglund et al. *Impact of telephone nursing education program for equity in healthcare*	Investigate if and how an educational intervention can improve awareness of equity in healthcare among telephone nurses	Telephone nurses and education	Nursing education	Quasi‐experimental
2017	Canada	Blanchet Garneau et al *Drawing on antiracist approaches toward a critical antidiscriminatory pedagogy for nursing*	Propose a critical antidiscriminatory pedagogy and thus translate social justice into nursing practice and education	Social justice and nursing	Nursing education	Theoretical
2017	Sweden	Holmström et al. *Nursing students' awareness of inequity in healthcare — An intersectional perspective*	Explore awareness of inequity in healthcare and the intersection between different structures of power among nursing students	Nursing students	Nursing education	Empirical – quantitative descriptive
2017	Canada	Kellett & Fitton *Supporting transvisibility and gender diversity in nursing practice and education*: *embracing cultural safety*	Raise awareness about the problems inherent to transinvisibility and propose interventions to increase the recognition of gender diversity in nursing education and practice	Transvisibility and trans gender clients	Nursing education and working conditions of nurses	Theoretical
2018	US	Damaskos et al. *Intersectionality and the LGBT* patient with cancer	Discuss cancer risk factors, health care access and treatment for lesbian, gay, bisexual, and transgender patients	LGBT patients with cancer	Oncology nursing	Review
2018	Canada	Elliott et al. *A focused ethnography of nursing team culture and leadership on a transitonal care unit*	Explore how a new staffing model impact the team dynamics within the socio‐political and sociocultural context of a transitional care unit	Nurses working environment and teamwork	Transitional care nursing	Empirical – qualitative
2018	US	Wesp et al. *An Emancipatory Approach to Cultural Competency*: *The Application of Critical Race, Postcolonial, and Intersectionality Theories*	Critique Guidelines regarding Culturally Competent Care	Marginalized groups and nurses cultural competency	All nursing	Theoretical
2019	New Zealand	Aspinall et al. *Intersectionality and Critical Realism*: *A Philosophical Framework for Advancing Nursing Leadership*	To develop an approach that addresses how the multiple social positions nurses hold an impact on their opportunities to develop as leaders	Nurses as leaders	Work environment of nurses	Theoretical
2019	Canada	Campbell et al. *Nurse‐family partnership and geography*: *an intersectional perspective*	Explore the influence of geography on the delivery of the public health program Nurse‐Family Partnership.	Nurse‐family partnership, maternal/child health and geography	Community nursing	Empirical – qualitative
2019	Canada	Clark et al. *Applying Critical Race Feminism and Intersectionality to Narrative Inquiry. A Point of Resistance for Muslim Nurses Donning a Hijab*	Explore racism within nursing and build a case for examining the experiences of Muslim nurses donning a hijab	Female Muslim nurses donning a hijab	Work environment of nurses	Theoretical
2019	US	DeWilde et al. *Structural Stress and Otherness*: *How Do They Influence Psychological Stress?*	Explore the impact of structural stressors and otherness on psychological stress. Determine if the cultural distress model aligns with definitions of culture among the participants.	Cultural distress and psychological stress	Hospital outpatient nursing	Empirical ‐ quantitative
2019	US	Engelman et al. *State of the Profession The Landscape of Disability Justice, Health Inequities, and Access for Patients With Disabilities*	Provide an overview of disparities faced by people with disabilities and recommendations for nursing curriculum and practice	People with disabilities	All nursing Nursing education	Theoretical
2019	US	Fitzgerald & Campinha‐Bacote *An Intersectionality Approach to the Process of Cultural Competemility – Part II*	By using an intersectionality approach, offer strategies that nurses and other healthcare professionals can use to challenge and address inequalities.	Nursing practice, organization and cultural humility	All nursing	Theoretical
2019	Canada	Henriquez, et al. *It's Complicated*: *Improving Undergraduate Nursing Students' Understanding Family and Care of LGBTQ Older Adults*.	Articulate a teaching approach and methodology of an unfolding LGBTQ family case study for undergraduate nursing students.	Undergraduate Nursing Students and LGBTQ Older Adults	Nursing education	Quasi‐experimental
2019	Canada	Reimer Kirkham *Complicating nursing's views on religion and politics in healthcare*.	Complicate nursing's views on religion and politics in healthcare	Nursing's views on religion and politics	All nursing	Theoretical
2019	Canada	Straus & Brown *The potential contribution of critical theories in healthcare transition research and practice*	The aim is to examine the potential contributions of intersectionality and critical discourse analysis to healthcare transition research and practice	Youth transitioning from childhood to adulthood and paediatric to adult health care	Transition from paediatric to adult care	Theoretical
2019	Canada	Thandi & Browne *The social context of substance use among older adults*: *Implications for nursing practice*.	To critically analyse the social context of substance use among older adults and to offer strategies to support the health of older adults experiencing problematic substance use.	Older adults with problematic substance use	All nursing	Theoretical
2019	US	Wardlaw & Shambley‐Ebron *Co‐cultural Communicative Practices of African American Women Seeking Depression Care*.	Explore the co‐cultural communicative practices that African American women use when seeking depression care	African‐ American women with depression	Mental health nursing	Empirical – qualitative
2020	US	Armour‐Burton & Etland *Black Feminist Thought*: *A Paradigm to Examine Breast Cancer Disparities*.	Examine how the intersection of race, gender, and class influences mental and physiological well‐being among African American Women with breast cancer	African American Women with cancer	Community nursing	Empirical – qualitative
2020	US	Griswold & Pagano‐Therrien *Women Living With HIV in High Income Countries and the Deeper Meaning of Breastfeeding Avoidance*: *A Metasynthesis*.	Describe social and emotional experiences of infant feeding for women living with HIV in high‐income countries and raise ethical considerations surrounding the clinical recommendation to avoid breastfeeding.	Breastfeeding women living with HIV in high‐income countries	All nursing	Review
2020	Australia	Ogrin et al. *The inter‐relationship of diversity principles for the enhanced participation of older people in their care*: *a qualitative study*.	Explore how five diversity principles are considered by older people	Older people	Home care nursing	Empirical – qualitative
2020	UK	Qureshi et al. *British South Asian male nurses' views on the barriers and enablers to entering and progressing in nursing careers*.	Ascertain British South Asian male nurses' views on the barriers and enablers to entering and progressing in nursing education and careers.	British South Asian male nurses	Work environment of nurses	Empirical – qualitative
2020	New Zealand	Aspinall et al. *The impact of intersectionality on nursing leadership, empowerment and culture*: *A case study exploring nurses and managers' perceptions in an acute care hospital in Aotearoa, New Zealand*.	Exploring intersectionality and nursing leadership in the context of the social environment	Nurses and managers in an acute care hospital	Work environment of nurses	Empirical – qualitative
2020	US	Weitzel et al. *The Role of Nurses as Allies Against Racism and Discrimination*: *An Analysis of Key Resistance Movements of Our Time*.	Offer nurses new epistemologies informed by intersectionality, critical race theory, and historical trauma to use in practice	Nurses' role in relation to marginalized populations	All nursing	Theoretical
2021	US	Burger et al. *Reproductive justice and black lives*: *A concept analysis for public health nursing*	To analyse the concept of reproductive justice as used in peer‐reviewed publications with the aim of reframing black maternal health in public health nursing	Black maternal health	Public health nursing	Review
2021	US	Crooks et al. *Black Female Sexuality*: *Intersectional Identities and Historical Contexts*	Explain how a secondary analysis of a study about Black female sexual behaviour supports intersectionality theory, describe an expanded model of intersectionality theory and discuss implications for nursing	Sexual health of Black women	All nursing	Empirical – qualitative secondary analysis
2021	US	Ramos et al. *Intersectional Effects of Sexual Orientation Concealment, Internalized Homophobia, and Gender Expression on Sexual Identity and HIV Risk Among Sexual Minority Men of Colour*: *A Path Analysis*.	Examine how intersecting identities, in aggregate, contribute to HIV risk in sexual minority men	Minority men and HIV risk and gender expression	All nursing	Empirical – quantitative
2021	US	Webster et al. *The Concept of Vulnerability Among Black and Latina Transgender Women in the United States*.	Review the current body of knowledge on vulnerability among Black and Latina transgender women	Black and Latina transgender women	All nursing	Review
2021	US	Ruiz et al. *A Historical Analysis of the Impact of Hegemonic Masculinities on Sexual Assault in the Lives of Ethnic Minority Women*: *Informing Nursing Interventions and Health Policy*	Deepen the understanding of how hegemonic masculinity shapes minority women's experiences of sexual assault	Minority women's experiences of sexual assault	All nursing	Review
2021	US	Ruiz et al. *An integrative literature review and critical reflection of intersectionality theory*	Critically reflect on intersectionality by considering the semantic and structural consistency, generalizability, simplicity and complexity, and the utility and value to nursing science and practice	Utility of intersectionality theory in nursing	All nursing	Review
2022	Canada	Al‐Hamad et al. *The Potential of Merging Intersectionality and Critical Ethnography for Advancing Refugee Women's Health Research*	Outline the potential of using the blended theoretical approach in advancing refugee women's health research and to inform a particular methodological approach for nursing research and health care practice	Refugee women	All nursing	Theoretical
2022	US	Bergman et al. *Reframing Intersectional Stigma for a South African Context Integrating Tuberculosis, HIV and Poverty Stigma*	Reframe intersectionality by considering a new set of stigmatized identities and create a situation‐specific framework	Intersectional stigma in South Africa	All nursing	Theoretical
2022	Brasil	Souza & Tanaka *Healthcare*: *Action research with trans people living on the streets*.	To analyse the representations of healthcare provided to trans people living on the streets.	Trans people living on the streets	Community nursing	Empirical – qualitative
2022	US	Schmitt et al. *Adverse childhood experiences among previously homeless African American women*.	The aim was to examine the role adverse childhood experiences played in life course trajectories of previously homeless African American women.	African American women who have previously been homeless	All nursing	Empirical‐ qualitative
2022	US	Schoon & Krumwiede *A holistic health determinants model for public health nursing education and practice*.	Propose a model for population health assessment	Health determinants	Nursing education	Theoretical
2022	UK	Qureshi et al. *Understanding the disproportionate effects of COVID‐19 on nurses from ethnic minority backgrounds*	Define intersectionality and outline intersecting factors contributing to disproportionate effects of COVID‐19 on nurses from ethnic minority backgrounds	Nurses from ethnic minority backgrounds	Work environment of nurses	Theoretical

There was a collection of different settings: nursing education (*n* = 7) (e.g. Höglund et al., 2016), the work environment of nurses (*n* = 7) (e.g. Aspinall et al., 2019), mental health nursing (*n* = 5) (e.g. Benbow et al., 2011), community nursing (*n* = 4) (e.g. Van Herk et al., 2011), home care (*n* = 2) (e.g. Giesbrecht et al., 2015) and oncology nursing (*n* = 2) (e.g. Damaskos et al., 2018). For one study respectively, the settings were nursing homes, public health nursing, transition from paediatric to adult care, hospital outpatient nursing, transitional care and school nursing. The majority (*n* = 27) of the reviewed articles did not concern any specific setting but concerned all nursing (e.g. Wesp et al., 2018). An overview of settings can be seen in Table 1.

There was heterogeneity in the patient group/group/phenomenon of interest in focus in the studies. A large group of the studies focused on race, people with immigrant/refugee status or people of racial or ethnic minority (*n* = 20) (e.g. Caxaj & Berman, 2010; Clark & Saleh, 2019; Henderson, 1997), women and/or mothers (*n* = 18) (e.g. Benbow et al., 2011; McCall & Lauridsen‐Hoegh, 2014; Wardlaw & Shambley‐Ebron, 2019) and/or the nursing profession (*n* = 11) (e.g. Aspinall et al., 2021; Jones et al., 2009; Qureshi et al., 2020). Many of the studies focused on all three of these patient groups/groups/phenomena of interest. Other patient groups/groups/phenomena of interest in focus in the studies were persons with HIV (*n* = 5) (e.g. Chulach & Gagnon, 2013), LGBT and transgender persons (*n* = 5) (e.g. Webster, 2021), victims of intimate partner violence (*n* = 4) (e.g. Guruge, 2012), youth/adolescents (*n* = 4) (e.g. Shade et al., 2011) and older adults (*n* = 3) (e.g. Ogrin et al., 2020). A few articles did not concern any specific patient group/group/phenomenon, but focused on research or the nursing discipline (*n* = 5) (e.g. Choby & Clark, 2014). An overview of patient groups/groups/phenomena of interest in focus in the studies can be seen in Table 1.

### 4.2 Definitions of intersectionality

Common references for intersectionality in the included studies were, for example, Hancock (2007), Hankivsky (2012), Collins (2000) and Crenshaw (1991). In the included studies, intersectionality was defined as a theoretical or refractory lens (e.g. Benbow et al., 2011; Reimer‐Kirkham, 2014; Van Herk et al., 2011), a tool (Kelly, 2011), a framework (e.g. Crooks et al., 2021; Guruge, 2012; Holmgren et al., 2014), a concept (e.g. Aspinall et al., 2021), a means of explaining (e.g. Jones et al., 2009), a perspective (e.g. Saarnio et al., 2012), a research method (e.g. Clark & Saleh, 2019; Crooks et al., 2021) and finally, a paradigm (e.g. Van Herk et al., 2011). A few studies did not clearly define intersectionality (e.g. Elliott et al., 2018; Henriquez et al., 2019; Qureshi et al., 2020).

There was generally an acknowledgment among the included studies on the interrelationship of processes at the micro, meso and macro levels in intersectional analysis, but the foci of attention differed between studies. For example, the oldest study by Henderson (1997), who was concerned with women's psychological development, focused on the micro level and argued that intersectionality could help us understand identity formation. Identity formation was also the subject of the paper by Shade et al. (2011), which discussed the identities available for adolescent boys in hypermasculine spaces such as criminal gangs and juvenile incarceration. Several studies explicitly mentioned processes at the micro, meso and macro levels (e.g. Blanchet Garneau et al., 2018; Choby & Clark, 2014; Guruge & Khanlou, 2004). Griswold and Pagano‐Therrien (2020) argued that intersectionality referred to how ‘social and political landscapes’ intersect with social locations, in their case, gender.

Intersectionality was described as linking to power struggles described as oppression (e.g. Wardlaw & Shambley‐Ebron, 2019), forces (e.g. Thandi & Browne, 2019), subordination (e.g. Kelly, 2009), inequalities and injustice (e.g. Damaskos et al., 2018), discrimination (e.g. Green, 2013), disenfranchisements (e.g. Choby & Clark, 2014), marginalization (e.g. Hall & Carlson, 2016), hierarchies and inclusion and exclusion (e.g. Höglund et al., 2016). Sometimes specific structures of power were called out as sexism, racism, colonialism (Henderson, 1997), ageism and classism (Guruge, 2012), and seen as being mutually constructed (e.g. Cuesta & Rämgård, 2016). The power struggles supposedly operated on micro, meso and macro levels as there were ‘multiple sites’ for them to play out (Guruge, 2012), for example, in the therapeutic encounter between the patient and the nurse (Van Herk et al., 2011).

Intersectionality was described as an anti‐reductionist concept (e.g. Giesbrecht et al., 2015) that aimed to capture the complexity of people's lives and experiences and put illness and health into context (e.g. Campbell et al., 2020; Reimer‐Kirkham, 2014). At the centre of analysis was what was described as social categories (e.g. Giesbrecht et al., 2015), social locations (e.g. Straus & Brown, 2021), social positions and relationships (e.g. Elliott et al., 2018) categories of difference (e.g. Clark & Saleh, 2019), dimensions of social life (e.g. Höglund et al., 2016), being ‘other’ (e.g. Hall & Carlson, 2016), social gradients (e.g. Kellett & Fitton, 2017), characteristics (e.g. Ogrin et al., 2020) or social divisions (e.g. Campbell et al., 2020). Such categories of interest were gender, race, age, sexual orientation and identity, ethnicity, culture, socioeconomic status, social class and ableness (e.g. Van Herk et al., 2010). These were all dynamic constructions in the sense that their meaning changed with time and place (e.g. Holmgren et al., 2014).

Van Herk et al. (2010) described race and gender as not being separate categories but as ‘interrelated and entangled’, i.e. they interact and intersect (e.g. Höglund et al., 2016), are indivisible (e.g. Wardlaw & Shambley‐Ebron, 2019) and interconnected (e.g. Damaskos et al., 2018). Identities were described as multiple and overlapping (e.g. Damaskos et al., 2018), simultaneous (e.g. Fitzgerald & Campinha‐Bacote, 2019), multiplicative (e.g. Kelly, 2009) and what Riemer‐Kirkham (2014, 250) described as a ‘hybrid’. Thus, the combination of two or more categories was suggested to invoke forms of oppression and experiences that were distinct from any of them standing alone (Green, 2013). Furthermore, no single category could or should be privileged according to several of the inuded studies (e.g. Henderson, 1997; Ogrin et al., 2020; Reimer‐Kirkham, 2014; Van Herk et al., 2011). Intersectionality was portrayed as capturing the nuances of individualized experiences (e.g. Hall & Carlson, 2016), and it was pointed out that there were differences among the group ‘women’ as there are diverse and shared experiences (e.g. Chulach & Gagnon, 2013; Guruge, 2012; Henderson, 1997).

### 4.3 Arguments for applying intersectionality in nursing research

Arguments for applying the concept of intersectionality were often to be able to consider the multiple natures of identities (e.g. Guruge & Khanlou, 2004), social locations (e.g. Giesbrecht et al., 2015; Straus & Brown, 2021) or categories such as race, gender and class (e.g. Ogrin et al., 2020), and migration (e.g. Guruge et al., 2010). Some studies argued that intersectionality was useful for analysing how disadvantages intersect with a specific phenomenon such as geographical contexts (Campbell et al., 2020) or religious signifiers such as the hijab (Clark & Saleh, 2019).

Several studies argued that intersectionality was useful for addressing issues concerning oppression and power (e.g. Aspinall et al., 2021; Griswold & Pagano‐Therrien, 2020; Holmgren et al., 2014). For example, by raising awareness and strengthening the understanding of how intersecting factors (e.g. Höglund et al., 2016; Thandi & Browne, 2019), social determinants (e.g. Caiola et al., 2014; Damaskos et al., 2018) or power structures (e.g. Holmström et al., 2017; Ruiz, Luebke, Hawkins, et al., 2021; Ruiz, Luebke, Klein, et al., 2021), contributed to inequities in health and affect health care delivery or how identity‐based structural oppressions functioned within an organization (e.g. Aspinall et al., 2019, 2021; Saarnio et al., 2012). Two studies specifically pointed out intersectionality as a means to investigate oppression and corresponding acts of resistance (Benbow et al., 2011; Caxaj & Berman, 2010).

Intersectionality was argued to enhance concepts or theories such as Orem's conditioning factors (Green, 2013), the concept of marginalization (Hall & Carlson, 2016), cultural safety (Kellett & Fitton, 2017), perspectives on cultural competency (Fitzgerald & Campinha‐Bacote, 2019; Wesp et al., 2018) and feminist theories of women's psychological development (Henderson, 1997). Adding to this, intersectionality was argued to inform narrow biomedical conceptualizations of health (Kelly, 2009), link ethical considerations and social justice concerning health disparities (Rogers & Kelly, 2011), inform understandings of religions and spirituality or politics (Reimer‐Kirkham, 2014, 2019), or simply forwarding analyses of structure and domination (Ruiz, Luebke, Hawkins, et al., 2021; Ruiz, Luebke, Klein, et al., 2021) or health disparities in nursing (Crooks et al., 2021). It was also put forward as a way to understand that nurses may be in positions of privilege and oppression at the same time depending on social conditions (Blanchet Garneau et al., 2018).

Several studies argued that intersectionality was suitable for understanding health‐related experiences and/or positions of specific groups. For example, African American women (Wardlaw & Shambley‐Ebron, 2019), HIV‐positive pregnant refugee women (Chulach & Gagnon, 2013), women experiencing intimate partner violence (Kelly, 2011), Aboriginal mothers (Van Herk et al., 2010), people with disabilities (Engelman et al., 2019) or American African women with breast cancer (Armour‐Burton & Etland, 2020).

In some studies, the arguments for applying intersectionality were not clearly articulated but rather explained by stating the premises of the theory/concept (DeWilde et al., 2019; Elliott et al., 2018; Jones et al., 2009; McCall & Lauridsen‐Hoegh, 2014). While most studies advocated for using intersectionality in nursing, a few pointed to difficulties or shortcomings with its application. These studies highlighted the ambiguity and inconsistency of the definition or application of intersectionality (e.g. Aspinall et al., 2019; Ruiz, Luebke, Hawkins, et al., 2021; Ruiz, Luebke, Klein, et al., 2021), the philosophical and practical problems of categorizing social groups (Kelly, 2009) and the difficulties in applying the concept concerning the method and measurements of outcomes (DeWilde et al., 2019; Green, 2013).

### 4.4 Author‐identified implications of applying intersectionality in nursing research

The author‐identified implications of applying intersectionality in nursing research were grouped into four categories: for research (e.g. how the application could enable the advancement of knowledge and theory within nursing research), for practice (e.g. how the application could influence the interaction between nurses and patients as well as nurses' career paths), for education (e.g. how the application could endorse pedagogical approaches that increase awareness of privilege, oppression, and the experiences of diverse groups among nursing students) and for society (e.g. how the application could emphasize concerns surrounding injustice and health disparities from a structural perspective within society).

#### 4.4.1 For research

The authors of the included studies identified several implications of applying intersectionality for research. Commonly, intersectionality was understood as allowing nursing research to advance the understanding of the role that social context and social processes played in influencing health and well‐being in different groups of people (e.g. Caxaj & Berman, 2010). For example, the understanding of how mechanisms and effects of social determinants influenced healthcare (Damaskos et al., 2018) or health interventions for different groups of people (e.g. Choby & Clark, 2014), or how other conditioning factors linked to race, gender and class (Armour‐Burton & Etland, 2020) affect health outcomes and how people experienced and dealt with their health in relation to these mechanisms and effects (Giesbrecht et al., 2015; Green, 2013). Intersectionality was also suggested to advance development of existing theories within nursing, for example, Orem's theory on conditioning factors (Green, 2013), theoretical understanding of religious and spiritual traditions (Reimer‐Kirkham, 2014), conceptual understanding of adolescent fatherhood (Shade et al., 2011), vulnerability (Webster, 2021) and the experiences of vulnerable groups (Saarnio et al., 2012).

#### 4.4.2 For practice

Several studies argued that nurses have a unique position to advocate for the health needs of populations and promote social justice in healthcare (e.g. Benbow et al., 2011; Blanchet Garneau et al., 2018). Intersectionality was suggested to promote a better understanding among health professionals of how structural issues could impact peoples' decisions regarding health and give a shared understanding of which healthcare goals are realistic and should be prioritized (Caiola et al., 2014). Advancement in knowledge on intersecting factors and health outcomes was suggested to re‐direct the focus of nursing practice (Armour‐Burton & Etland, 2020). For example, by helping nurses to better understand structural causes for why some persons are ill‐equipped to prioritize their health (Thandi & Browne, 2019) and refrain from blaming individuals for risky behaviours (Crooks et al., 2021). By understanding and respecting the cultural and societal milieu of marginalized communities, it was argued that the nurse could provide compassionate care (Schmitt et al., 2022). Intersectionality was suggsted to have implications for the therapeutic encounter between nurses and patients by acknowledging its inherent power dynamics (Van Herk et al., 2010) and by guiding nurses in communicating more respectfully and avoiding pejorative labels (Ogrin et al., 2020; Thandi & Browne, 2019). Knowledge informed by intersectionality was argued to be a helpful tool to act on injustice in practice and create inclusive healthcare institutions (Reimer‐Kirkham, 2019; Weitzel et al., 2020) and support nurses in self‐reflection regarding their own biases and how to avoid stigmatizing patients (Ogrin et al., 2020; Thandi & Browne, 2019). Some authors suggested that intersectional nursing research should combine scholarship with activism (Caiola et al., 2014; Souza & Tanaka, 2021). Finally, intersectionality was suggested as useful for understanding how intersecting social identities could, for example, prevent some nurses from being leaders (Aspinall et al., 2019, 2021) or how gender and ethnicity influence the decision to enter the nursing field (Qureshi et al., 2020).

#### 4.4.3 For education

Several authors argued that intersectionality could be applied in the educational field to promote, for example, antiracist education and critical antidiscriminatory pedagogical approaches (e.g. Blanchet Garneau et al., 2018; Caiola et al., 2014; Clark & Saleh, 2019). Some of the studies suggested training to enhance nursing students' awareness of the experiences of specific groups such as transgender persons (Webster, 2021) or people with disabilities (e.g. Green, 2013). Clark and Saleh (2019) pointed out the underrepresentation of racialized and indigenous people among faculty members in institutional places of learning while Kellett and Fitton (2017) called for more trans visibility in academia through features such as gender‐neutral washrooms and allowing students to use their preferred pronouns. Adding to this, authors suggested that nursing education could engage the different communities as a source of knowledge, including the trans community (Kellett & Fitton, 2017) and older people (Ogrin et al., 2020). Intersectionality was also argued to inform nursing to reflect on issues of power and privilege (Wardlaw & Shambley‐Ebron, 2019) and help students become more aware of their social position and the privilege that comes with the nursing role (Crooks et al., 2021). One way this was implemented was by supporting discussions on health equity (Höglund et al., 2016).

#### 4.4.4 For society

Authors stated that intersectionality calls for social change (Kelly, 2009), as one must question (Elliott et al., 2018) and challenge injustice (Aspinall et al., 2019) upon discovering its prevalence. Henderson (1997) argued that intersectionality could illuminate how white women become complicit in racism by replicating images of themselves as nurturing and ethical. Intersectionality was also suggested to highlight the structural issues related to health, such as the need to increase opportunities for employment, housing and financial support (Webster, 2021). This could inform nursing by addressing social injustice (Reimer‐Kirkham, 2014). Finally, intersectionality was argued to shed light on nurse migration as an issue in need of a more gender‐centred approach to recruitment and policy where the well‐being of migrant nurses might be undermined through discrimination even when their financial situation is improved (Jones et al., 2009).

## 5 DISCUSSION

The application of intersectionality in nursing research has primarily emerged since 2010, originating from nations like Canada and the United States, and is non‐empirical. Our review identified a broad variation in the definition and use of intersectionality in nursing research. What constitutes an intersectional analysis is debated, and there is a broader lack of consensus on the definition of intersectionality (Bilge, 2013; Collins, 2015; Davis, 2008). Together, the studies included in this analysis cover all of the six fundamental ideas of intersectionality: complexity, power, inequality, relationality, social context and social justice (Collins & Bilge, 2016). They highlight the diversity present among various groups (e.g. women), the impact of power structures and relations on specific groups (e.g. trans persons), unequal access to opportunities and resources (e.g. among groups of nurses), the interrelation between social inequalities and systems of power (e.g. concerning race and gender) and the influence of social context on people's experiences (e.g. concerning migration). Ultimately, they advocate for equitable distribution of resources and opportunities related to healthcare. However, when considered as a whole, these studies demonstrate numerous approaches to defining intersectionality such as using it as a tool, lens, paradigm or method. Similarly, intersectionality is put forward to enable the study of multiple dimensions, but the units of analysis vary between the studies. Many of the cases in our review focus on units of analysis that are commonly centred in intersectional analyses such as identities (e.g. black women), categories (e.g. gender), processes of differentiation (e.g. gendering) and systems of domination (e.g. patriarchy) (Dhamoon, 2011), but several studies also focus on other aspects such as geographical contexts, being other and social gradients. For intersectionality—a borrowed theory—to enrich nursing research and issues relevant to nursing practice, it must be adapted to fit the nursing context (Risjord, 2010). For the promises of intersectionality to be realized, there is also a need for nursing research to make clear distinctions between levels of analysis and to provide robust and clear framing of what is intersecting and what this means in different cases (cf. Anthias, 2013).

A common argument among the studies for using intersectionality is to raise awareness and broaden the understanding of health‐related experiences and inequities in health and healthcare delivery. Most of the studies in this review focus on marginalized groups. Several of the studies in this review do not discuss how, when or where intersecting processes of discrimination/subordination are significant for the specific individuals or groups of study. Rather, they assess such a process by the gender, race, sexuality, etc., of these individuals and groups. A common pitfall in making intersectional analyses is listing these categories that intersect rather than analytically attending to them (Anthias, 2013). Such assumptions risk treating identities or categories as given and rigid rather than constructed. Thereby, they risk an over‐determinate understanding of difference and re‐producing existing hegemonies (Dhamoon, 2011). This, we argue, also risks leading to victimization. Benbow et al. (2011) and Caxaj and Berman (2010) specifically point out resilience and acts of resistance by marginalized groups as one way to counteract this.

Nursing studies that apply intersectionality will benefit from reviewing how analytical assumptions are empirically addressed and operationalized. Rather than searching for defining oppression and power structures with specific groups and their characteristics as a starting point, research should focus on the mechanisms that produce results like oppression and discrimination. This would allow for analysis of the functions of the mechanisms of power and oppression rather than attributing its negative consequences to specific groups.

Only a few studies point to difficulties or shortcomings with operationalizing the concept of intersectionality. Nursing studies should take a nuanced and explicit position concerning intersectionality. Hence, when conducting studies adopting intersectionality, there should also be room for addressing the approach's shortcomings. By ‘explicit position’ we mean that researchers should aim to be clear about what they refer to as intersectionality and how their use of the concept is linked to theorists and previous research.

Finally, this review points to the limited number of empirical nursing studies that address intersectional analysis. Previous research has pointed to ambivalence in how intersectionality can be integrated with empirical projects (Hancock, 2016). The question of how to design empirical studies in nursing that conducts intersectional analysis is something that needs to be further explored in future research. There is a need for more studies that effectively combine theoretical insights from intersectionality with empirical observations from the nursing field.

### 5.1 Strengths and limitations

To the best of our knowledge, this is the first systematic review of using intersectionality in nursing. This study is warranted by the need for theory development in nursing, especially concerning so‐called ‘borrowed theories’ (Risjord, 2010). Such theory development will benefit from a description of the myriad of ways that intersectionality is adopted in nursing research. The review followed an extensive search strategy to cover a maximum amount of nursing research. For example, the full text of all studies published in a nursing journal were screened regardless of whether the abstract or title mentioned nursing. We did not include grey literature, theses, or studies not published in English, but regard the included studies as representative of the field of peer‐reviewed nursing studies applying intersectionality.

The rigour of any systematic review should be addressed, and researchers should carefully consider, justify and adhere to their choice of methods for doing a literature review (Aveyard & Bradbury‐Jones, 2019). In this paper, a systematic review was deemed appropriate as we aimed to identify, appraise and synthesize all nursing research adopting intersectionality. Due to the heterogeneity of the design of the included studies, we did not conduct a meta‐analysis. Instead, we conducted a narrative synthesis by deductively and iteratively summarizing the data from the articles. A critique of narrative synthesis is its lack of transparency. To increase transparency, the approach taken in this paper is described in the method section. Nevertheless, there is always a risk of bias when conducting a narrative synthesis. This should be taken into consideration. To meet the aim of this systematic review we included both empirical studies and review articles. This resulted in that in some cases results of the original article were repeated in the included review article introducing a risk of bias. However, since this narrative synthesis did not quantify findings but describe the variation in how intersectionality has been applied in nursing research the risk of such bias is minimized. To assess the overall methodological quality of the included studies, we conducted a quality appraisal, but we did not exclude articles based on methodological quality, which could be interpreted as a possible limitation (see Aveyard & Bradbury‐Jones, 2019). However, the aim of this review was to identify, appraise and synthesize all available research articles that apply intersectionality in nursing, not solely studies deemed to be of good quality.

## 6 CONCLUSION

Intersectionality can help develop understandings of the workings of power and oppression in nursing research by, for example, moving beyond the patient‐dyad perspective and individual‐focused frameworks in nursing to address peoples' social context, which has been called for by previous studies (Tengelin et al., 2019; Thurman & Pfitzinger‐Lippe, 2017). This review identifies a myriad of ways to define and use intersectionality in nursing research. While diversity per se is not a problem, there is a need for nursing research to provide robust and clear framing of how intersectionality is understood, how analytical assumptions are addressed and how the concept is empirically operationalized. Finally, there is a lack of nursing studies that combine theoretical insights from intersectionality with empirical observations. Therefore, there is a need for more empirical research that effectively adopts the concept of intersectionality to develop the understanding of the workings of health inequities and issues of power and domination in nursing.

## FUNDING INFORMATION

No funding was received for the work with this paper.

## CONFLICT OF INTEREST STATEMENT

Nothing to declare.

## ETHICS STATEMENT

Ethical approval was not required as the systematic review was performed on published data.

## Supporting information


Data S1.

## Data Availability

Data sharing not applicable to this article as no datasets were generated or analysed during the current study
