# Supplementary material for: Intersectionality in nursing research: A systematic review
Source: Nurs Open. 2023 Oct 5;10(12):7509–27. doi: 10.1002/nop2.2021 (PMC10643838; doi:10.1002/nop2.2021)
Supplement: Supplementary file 1 — Data S1. [file NOP2-10-7509-s001.docx]

SUPPLEMENTARY INFORMATION

**Table 1. PRISMA 2020 checklist (The Preferred Reporting Items for Systematic Reviews and Meta-Analyses) (Page et al., 2021)**

| **Section and Topic** | **Item #** | **Checklist item** | **Location where item is reported** |
| --- | --- | --- | --- |
| **TITLE** | | |  |
| Title | 1 | Identify the report as a systematic review. | Title |
| **ABSTRACT** | | |  |
| Abstract | 2 | See the PRISMA 2020 for Abstract checklist. | Abstract |
| **INTRODUCTION** | | |  |
| Rationale | 3 | Describe the rationale for the review in the context of existing knowledge. | Introduction |
| Objectives | 4 | Provide an explicit statement of the objective(s) or question(s) the review addresses. | Background |
| **METHODS** | | |  |
| Eligibility criteria | 5 | Specify the inclusion and exclusion criteria for the review and how studies were grouped for the syntheses. | Study selection process  Data synthesis |
| Information sources | 6 | Specify all databases, registers, websites, organizations, reference lists, and other sources searched or consulted to identify studies. Specify the date when each source was last searched or consulted. | Search methods |
| Search strategy | 7 | Present the full search strategies for all databases, registers, and websites, including any filters and limits used. | Search methods  Supplementary material |
| Selection process | 8 | Specify the methods used to decide whether a study met the inclusion criteria of the review, including how many reviewers screened each record and each report retrieved, whether they worked independently, and if applicable, details of automation tools used in the process. | Study selection process |
| Data collection process | 9 | Specify the methods used to collect data from reports, including how many reviewers collected data from each report, whether they worked independently, any processes for obtaining or confirming data from study investigators, and if applicable, details of automation tools used in the process. | Data extraction  Data synthesis |
| Data items | 10a | List and define all outcomes for which data were sought. Specify whether all results that were compatible with each outcome domain in each study were sought (e.g., for all measures, time points, analyses), and if not, the methods used to decide which results to collect. | Data extraction  Data synthesis |
|  | 10b | List and define all other variables for which data were sought (e.g., participant and intervention characteristics, funding sources). Describe any assumptions made about any missing or unclear information. | Data extraction  Data synthesis |
| Study risk of bias assessment | 11 | Specify the methods used to assess the risk of bias in the studies, including details of the tool(s) used, how many reviewers assessed each study, and whether they worked independently, and if applicable, details of automation tools used in the process. | Quality appraisal |
| Effect measures | 12 | Specify for each outcome the effect measure(s) (e.g., risk ratio, mean difference) used in the synthesis or presentation of results. | N/A |
| Synthesis methods | 13a | Describe the processes used to decide which studies were eligible for each synthesis (e.g., tabulating the study intervention characteristics and comparing against the planned groups for each synthesis (item #5)). | Data extraction  Data synthesis  Table 1 |
|  | 13b | Describe any methods required to prepare the data for presentation or synthesis, such as handling missing summary statistics or data conversions. | N/A |
|  | 13c | Describe any methods used to tabulate or visually display the results of individual studies and syntheses. | N/A |
|  | 13d | Describe any methods used to synthesize results and provide a rationale for the choice(s). If a meta-analysis was performed, describe the model(s) and method(s) to identify the presence and extent of statistical heterogeneity, as well as the software package(s) used. | Data synthesis |
|  | 13e | Describe any methods used to explore the possible causes of heterogeneity among study results (e.g., subgroup analysis, meta-regression). | N/A |
|  | 13f | Describe any sensitivity analyses conducted to assess robustness of the synthesized results. | N/A |
| Reporting bias assessment | 14 | Describe any methods used to assess the risk of bias due to missing results in a synthesis (arising from reporting biases). | Quality appraisal |
| Certainty assessment | 15 | Describe any methods used to assess certainty (or confidence) in the body of evidence for an outcome. | Quality appraisal |
| **RESULTS** | | |  |
| Study selection | 16a | Describe the results of the search and selection process, from the number of records identified in the search to the number of studies included in the review, ideally using a flow diagram. | Results  Figure 1 |
|  | 16b | Cite studies that might appear to meet the inclusion criteria but were excluded, and explain why they were excluded. |  |
| Study characteristics | 17 | Cite each included study and present its characteristics. | Table 1 |
| Risk of bias in studies | 18 | Present assessments of risk of bias for each included study. | N/A |
| Results of individual studies | 19 | For all outcomes, present for each study: (a) summary statistics for each group (where appropriate), and (b) an effect estimate and its precision (e.g., confidence/credible interval), ideally using structured tables or plots. | Table 1: characteristics of individual studies |
| Results of syntheses | 20a | For each synthesis, briefly summarize the characteristics and risk of bias among contributing studies. | N/A |
|  | 20b | Present results of all statistical syntheses conducted. If meta-analysis was done, present for each the summary estimate and its precision (e.g., confidence/credible interval) and measures of statistical heterogeneity. If comparing groups, describe the direction of the effect. | N/A |
|  | 20c | Present results of all investigations of possible causes of heterogeneity among study results. | N/A |
|  | 20d | Present results of all sensitivity analyses conducted to assess the robustness of the synthesized results. | N/A |
| Reporting biases | 21 | Present assessments of risk of bias due to missing results (arising from reporting biases) for each synthesis assessed. | N/A |
| Certainty of evidence | 22 | Present assessments of certainty (or confidence) in the body of evidence for each outcome assessed. | N/A |
| **DISCUSSION** | | |  |
| Discussion | 23a | Provide a general interpretation of the results in the context of other evidence. | Discussion |
|  | 23b | Discuss any limitations of the evidence included in the review. | Strengths and limitations |
|  | 23c | Discuss any limitations of the review processes used. | Strengths and limitations |
|  | 23d | Discuss implications of the results for practice, policy, and future research. | Conclusion |
| **OTHER INFORMATION** | | |  |
| Registration and protocol | 24a | Provide registration information for the review, including register name and registration number, or state that the review was not registered. | N/A |
|  | 24b | Indicate where the review protocol can be accessed or state that a protocol was not prepared. | N/A |
|  | 24c | Describe and explain any amendments to information provided at registration or in the protocol. | N/A |
| Support | 25 | Describe sources of financial or non-financial support for the review and the role of the funders or sponsors in the review. | Funding statement |
| Competing interests | 26 | Declare any competing interests of review authors. | Conflict of interest |
| Availability of data, code, and other materials | 27 | Report which of the following are publicly available and where they can be found: template data collection forms, data extracted from included studies, data used for all analyses, analytic code, and any other materials used in the review. | N/A |

**Table 2. Records identified through database search (PubMed and CINAHL)**

| **DATABASES** | **Search string** | **n** = |
| --- | --- | --- |
| CINAHL | (intersectional* AND nurs*) | 207 |
| PubMed | (intersectional* AND nurs*) | 197 |
| Total |  | 404 |
| Duplicates |  | 73 |
| Total records screened |  | 331 |

**Table 3. Quality assessment of included studies using the critical appraisal tools from the Joanna Briggs Institute (JBI, 2022) according to study design: Text and opinion papers (McArthur et al., 2015). For quality assessment questions, see table notes.**

| **Year** | **Author** | **Q1** | **Q2** | **Q3** | **Q4** | **Q5** | **Q6** |
| --- | --- | --- | --- | --- | --- | --- | --- |
| 1997 | Henderson | Yes | Unclear | Yes | Yes | Yes | N/A |
| 2004 | Guruge & Khanlou | Yes | Unclear | Yes | Yes | Yes | N/A |
| 2009 | Kelly | Yes | Unclear | Yes | Yes | Yes | N/A |
| 2011 | Shade et al. | Unclear | Unclear | Yes | Yes | Yes | N/A |
| 2011 | Rogers & Kelly | Yes | Unclear | Yes | Yes | Yes | N/A |
| 2011 | Kelly | Unclear | Unclear | Yes | Yes | Yes | N/A |
| 2012 | Guruge | Yes | Yes | Yes | Yes | Yes | Yes |
| 2013 | Choby & Clark | Yes | Unclear | Yes | Yes | Yes | N/A |
| 2013 | Chulach & Gagnon | Unclear | Unclear | Yes | Yes | Yes | N/A |
| 2013 | Green | Unclear | Unclear | Yes | Yes | Yes | N/A |
| 2014 | Caiola et al. | Unclear | Unclear | Yes | Yes | Yes | N/A |
| 2014 | Reimer-Kirkham | Yes | Unclear | Yes | Yes | Yes | N/A |
| 2016 | Hall & Carlson | Unclear | Unclear | Yes | Yes | Yes | N/A |
| 2017 | Blanchet Garneau et al. | Yes | Unclear | Yes | Yes | Yes | N/A |
| 2017 | Kellet & Fitton | Unclear | Unclear | Yes | Yes | Yes | N/A |
| 2018 | Damaskos et al. | Unclear | Unclear | Yes | Yes | Yes | N/A |
| 2018 | Wesp et al. | Unclear | Unclear | Yes | Yes | Yes | N/A |
| 2019 | Aspinall et al. | Unclear | Unclear | Yes | Yes | Yes | N/A |
| 2019 | Clark et al. | Yes | Yes | Yes | Yes | Yes | N/A |
| 2019 | Engelman et al. | Yes | Unclear | Yes | Yes | Yes | N/A |
| 2019 | Fitzgerald & Campinha-Bacote | Unclear | Unclear | Yes | Yes | Yes | N/A |
| 2019 | Straus & Brown | Unclear | Unclear | Yes | Yes | Yes | Yes |
| 2019 | Reimer-Kirkham | Yes | Unclear | Yes | Yes | Yes | N/A |
| 2019 | Thandi & Browne | Unclear | Unclear | Yes | Yes | Yes | N/A |
| 2020 | Weitzel et al. | Yes | Unclear | Yes | Yes | Unclear | No |
| 2021 | Ruiz et al. | Yes | Yes | Yes | Yes | Yes | Yes |
| 2021 | Quershi et al. | Yes | Unclear | Yes | Yes | Unclear | Yes |
| 2021 | Webster | Yes | Unclear | Yes | Yes | Yes | Yes |
| 2022 | Al-Hamad et al. | Yes | Unclear | Yes | Yes | Yes | Yes |
| 2022 | Bergman et al. | Yes | Unclear | Yes | Yes | Yes | Yes |
| 2022 | Schoon & Krumweide | Yes | Unclear | Yes | Yes | Unclear | No |

**Notes**: Q1 = Is the source of the opinion clearly identified? Q2 = Does the source of opinion have standing in the field of expertise? Q3 = Are the interests of the relevant population the central focus of the opinion? Q4 = Is the stated position the result of an analytical process, and is there logic in the opinion expressed? Q5 = Is there reference to the extant literature? Q6 = Is any incongruence with the literature/sources logically defended? (JBI, 2022).

**Table 4. Quality assessment of included studies using the critical appraisal tools from the Joanna Briggs Institute (JBI, 2022) according to study design: Qualitative research (Lockwood et al., 2015). For quality assessment questions, see table notes.**

| **Year** | **Author** | **Q1** | **Q2** | **Q3** | **Q4** | **Q5** | **Q6** | **Q7** | **Q8** | **Q9** | **Q10** |
| --- | --- | --- | --- | --- | --- | --- | --- | --- | --- | --- | --- |
| 2009 | Guruge et al. | Yes | Yes | Yes | Yes | Yes | Yes | Unclear | Yes | Yes | Yes |
| 2010 | Caxaj & Berman | Yes | Unclear | Yes | Unclear | Unclear | Yes | Yes | Unclear | Yes | Yes |
| 2010 | Van Herk et al. | Yes | Yes | Yes | Yes | Yes | Yes | Unclear | Yes | Yes | Yes |
| 2011 | Benbow et al. | Yes | Unclear | Yes | Yes | Yes | Yes | Yes | Yes | Yes | Yes |
| 2011 | Van Herk et al. | Yes | Yes | Yes | Yes | Yes | Yes | Unclear | Yes | Yes | Yes |
| 2012 | Saarnio et al. | Unclear | Yes | Yes | Yes | Yes | Unclear | Yes | Yes | Yes | Yes |
| 2013 | Giesbrecht et al. | Yes | Yes | Yes | Yes | Yes | Unclear | Unclear | Yes | Yes | Yes |
| 2014 | Holmgren et al. | Yes | Unclear | Yes | Yes | Yes | Yes | Yes | Yes | Yes | Yes |
| 2016 | Cuesta & Rämgård | Yes | Yes | Yes | Yes | Yes | Unclear | No | Yes | Yes | Yes |
| 2018 | Elliott et al. | Yes | Yes | Yes | Yes | Yes | Unclear | Unclear | Yes | Yes | Yes |
| 2019 | Campbell et al. | Yes | Yes | Yes | Yes | Yes | Yes | Unclear | Yes | Yes | Yes |
| 2019 | Wardlaw & Shambley-Ebron | Yes | Yes | Yes | Yes | Yes | Unclear | Unclear | Yes | Yes | Yes |
| 2020 | Armour-Burton & Etland | Yes | Yes | Yes | Yes | Yes | Yes | Yes | Yes | Yes | Yes |
| 2020 | Ogrin et al. | Yes | Yes | Yes | Yes | Yes | Yes | Yes | Yes | Yes | Yes |
| 2020 | Qureshi et al. | Unclear | Unclear | Unclear | Unclear | Unclear | No | Yes | Yes | Yes | Yes |
| 2021 | Aspinall et al. | Yes | Unclear | Yes | Yes | Unclear | Yes | Yes | Yes | Yes | Yes |
| 2021 | Crooks et al. | Yes | Yes | Yes | Yes | Yes | Yes | Yes | Yes | Yes | Yes |
| 2022 | Schmitt et al. | Yes | Yes | Yes | Yes | Yes | Yes | Yes | Yes | Yes | Yes |
| 2022 | Souza & Tanka | Yes | Unclear | Yes | Yes | Unclear | Yes | No | Yes | Yes | Unclear |

**Notes:** Q1 = Is there congruity between the stated philosophical perspective and the research methodology? Q2 = Is there congruity between the research methodology and the research question or objectives? Q3 = Is there congruity between the research methodology and the methods used to collect data? Q4 = Is there congruity between the research methodology and the representation and analysis of data? Q5 = Is there congruity between the research methodology and the interpretation of results? Q6 = Is there a statement locating the researcher culturally or theoretically? Q7 = Is the influence of the researcher on the research and vice versa addressed? Q8 = Are participants and their voices adequately represented? Q9 = Is the research ethical according to current criteria or, for recent studies, is there evidence of ethical approval by an appropriate body? Q10 = Do the conclusions drawn in the research report flow from the analysis, or interpretation, of the data? (JBI, 2022).

**Table 5. Quality assessment of included studies using the critical appraisal tools from the Joanna Briggs Institute (JBI, 2022) according to study design: Systematic reviews and meta-synthesis (Aromataris et al., 2015). For quality assessment questions, see table notes.**

| **Year** | **Author** | **Q1** | **Q2** | **Q3** | **Q4** | **Q5** | **Q6** | **Q7** | **Q8** | **Q9** | **Q10** | **Q11** |
| --- | --- | --- | --- | --- | --- | --- | --- | --- | --- | --- | --- | --- |
| 2009 | Jones et al. | Yes | Yes | Yes | Yes | Yes | Unclear | Unclear | Unclear | Unclear | Yes | Yes |
| 2020 | Griswold & Pagano-Therrien | Yes | Yes | Yes | Yes | N/A | Yes | Yes | Yes | Unclear | N/A | N/A |
| 2021 | Burger et al. | Yes | Yes | Yes | Unclear | No | No | Yes | Yes | N/A | Yes | Yes |
| 2021 | Ruiz et al. | Yes | Yes | Yes | Yes | N/A | N/A | Yes | Yes | No | Yes | Yes |

**Notes:** Q1 = Is the review question clearly and explicitly stated? Q2 = Were the inclusion criteria appropriate for the review question? Q3 = Was the search strategy appropriate? Q4 = Were the sources and resources used to search for studies adequate? Q5 = Were the criteria for appraising studies appropriate? Q6 = Was critical appraisal conducted by two or more reviewers independently? Q7 = Were there methods to minimize errors in data extraction? Q8 = Were the methods used to combine studies appropriate? Q9 = Was the likelihood of publication bias assessed? Q10 = Were recommendations for policy and/or practice supported by the reported data? Q11 = Were the specific directives for new research appropriate? (JBI, 2022).

**Table 6. Quality assessment of included studies using the critical appraisal tools from the Joanna Briggs Institute (JBI, 2022) according to study design: Cross-sectional studies (Moola et al., 2020). For quality assessment questions, see table notes.**

| **Year** | **Author** | **Q1** | **Q2** | **Q3** | **Q4** | **Q5** | **Q6** | **Q7** | **Q8** |
| --- | --- | --- | --- | --- | --- | --- | --- | --- | --- |
| 2017 | Holmström et al. | Yes | Yes | N/A | Yes | N/A | N/A | Yes | Yes |
| 2019 | DeWilde et al. | Yes | Yes | Yes | Yes | N/A | N/A | Yes | Yes |
| 2021 | Ramos et al. | Yes | Yes | N/A | Yes | Yes | Yes | Yes | Yes |

**Notes:** Q1 = Were the criteria for inclusion in the sample clearly defined? Q2 = Were the study subjects and the setting described in detail? Q3 = Was the exposure measured in a valid and reliable way? Q4 = Were objective, standard criteria used for measuring the condition? Q5 = Were confounding factors identified? Q6 = Were strategies to deal with confounding factors stated? Q7 = Were the outcomes measured in a valid and reliable way, Q8 = Was an appropriate statistical analysis used? (JBI, 2022).

**Table 7. Quality assessment of included studies using the critical appraisal tools from the Joanna Briggs Institute (JBI, 2022) according to study design: Case reports (Moola et al., 2020). For quality assessment questions, see table notes.**

| **Year** | **Author** | **Q1** | **Q2** | **Q3** | **Q4** | **Q5** | **Q6** | **Q7** | **Q8** |
| --- | --- | --- | --- | --- | --- | --- | --- | --- | --- |
| 2013 | McCall & Lauridsen-Hoegh | Yes | Unclear | N/A | N/A | N/A | N/A | Yes | Yes |

**Notes:** Q1 = Were the patient’s demographic characteristics clearly described? Q2 = Was the patient’s history clearly described and presented as a timeline? Q3 = Was the current clinical condition of the patient on presentation clearly describedQ4 = Were diagnostic tests or assessment methods and the results clearly described? Q5 = Were the intervention(s) or treatment procedure(s) clearly described? Q6 = Was the post-intervention clinical condition clearly described? Q7 = Were adverse events (harms) or unanticipated events identified and described? Q8 = Does the case report provide takeaway lessons? (JBI, 2022).

**Table 8. Quality assessment of included studies using the critical appraisal tools from the Joanna Briggs Institute (JBI, 2022) according to study design: Quasi-experimental studies (Tufanaru et al., 2020).**  **For quality assessment questions, see table notes.**

| **Year** | **Author** | **Quality assessment tool** | **Q1** | **Q2** | **Q3** | **Q4** | **Q5** | **Q6** | **Q7** | **Q8** | **Q9** |
| --- | --- | --- | --- | --- | --- | --- | --- | --- | --- | --- | --- |
| 2016 | Höglund et al. | Quasi-experimental | Yes | Yes | Unclear | Yes | Yes | Unclear | Yes | Yes | Yes |
| 2019 | Henriquez et al. | Quasi-experimental | Yes | N/A | No | No | Yes | Yes | No | Yes | Yes |

**Notes:** Q1 = Is it clear in the study what is the ‘cause’ and what is the ‘effect’ (i.e., there is no confusion about which variable comes first)? Q2 = Were the participants included in any comparisons similar? Q3 = Were the participants included in any comparisons receiving similar treatment/care, other than the exposure or intervention of interest? Q4 = Was there a control group? Q5 = Were there multiple measurements of the outcome, both before and after the intervention/exposure? Q6 = Was follow-up complete and, if not, were differences between groups in terms of their follow-up adequately described and analyzed? Q7 = Were the outcomes of participants included in any comparisons measured in the same way? Q8 = Were outcomes measured in a reliable way? Q9 = Was appropriate statistical analysis used? (JBI, 2022).
